# The Influence of Varying Degrees of Enactability on the Enactment Effect in Action Memory During the Encoding and Retrieval Stages: A Study with Healthy Young Adults

**DOI:** 10.3390/bs16030438

**Published:** 2026-03-17

**Authors:** Hui Cao, Guangzheng Li

**Affiliations:** School of Education Science, Jiangsu Normal University, Xuzhou 221116, China

**Keywords:** action memory, enactment effect, motor information, degree of enactability, motor information reactivation

## Abstract

Whether the enactment effect benefits from motor information activation is a key concern in action memory; meanwhile, the degree of enactability may influence this activation. Accordingly, this study aims to examine the explanatory role of motor information reactivation in the enactment effect and to further clarify whether a certain degree of motor information activation is necessary for this effect to emerge. To this end, we manipulated the degree of enactability and separately investigated its impact on the enactment effect at the encoding stage (Experiment 1) and the retrieval stage (Experiment 2). Experiment 1 required participants to either silently read phrases or both silently read and physically enact the actions represented by the phrases during encoding. The research showed that an enactment effect was only observed for the high-enactability-phrases condition, but not for the low-enactability-phrases condition. Experiment 2 additionally required participants to either verbally recall (verbal retrieval) or verbally recall while simultaneously performing corresponding actions (enactment retrieval) during retrieval. The findings showed that under the verbal retrieval condition, the enactment effect was observed for the high-enactability-phrases condition but not for the low-enactability-phrases condition; under the enactment retrieval condition, the enactment effect was observed both for the high-enactability phrases and low-enactability-phrases conditions. Thus, motor information activation during encoding and retrieval is crucial for the enactment effect, which emerges only when motor information activation reaches a threshold, supporting and expanding motor information reactivation theory.

## 1. Introduction

In order to better understand or master knowledge, people often incorporate various actions while reading books or memorizing key points. In the laboratory, researchers primarily employ the subject-performed task (SPT) paradigm to investigate action memory (Cohen, 1981). In this paradigm, participants are presented with a series of phrases (e.g., “open a book”) and instructed to either perform the actions represented by the phrases while silently reading them (SPT) or simply read the phrases silently (verbal task, VT). Recall results show that memory performance under the SPT condition is significantly better than under the VT condition. This phenomenon is known as the SPT effect or the enactment effect (Engelkamp & Zimmer, 1985). Whether the research involves adult participants (Cohen, 1989; Engelkamp & Zimmer, 1997), young children (Badinlou et al., 2017; Kormi-Nouri et al., 2003), elderly participants (Schatz et al., 2011; Tuena et al., 2019), or participants such as children with autism (Grainger et al., 2017; Wojcik et al., 2011), a robust SPT effect has been consistently found (Roberts et al., 2022). However, regarding the mechanism underlying the enactment effect, no consensus has been reached. This study aims to delve deeply into the internal mechanism of the enactment effect.

The topic of how actions affect memory has been extensively studied not only in the realm of long-term memory but also in the field of working memory (Allen et al., 2020, 2023; Allen & Waterman, 2015; Li et al., 2022). Studies combining the working memory paradigm with the SPT paradigm have demonstrated that the enactment effect also exists within the scope of working memory, and this effect is mainly driven by the spatial-motoric coding activated during enactment (Jaroslawska et al., 2016, 2018; Waterman et al., 2017; Yang et al., 2015). Such research findings may help explain the emergence of action memory, but working memory and long-term memory are likely governed by multiple distinct mechanisms (e.g., Baddeley, 1966a, 1966b; Norris, 2017).

### 1.1. Processing Process Theory vs. Processing System Theory

Current research on the enactment effect primarily focuses on the mechanisms behind its occurrence. Explanations regarding the generative mechanism of the enactment effect can be broadly categorized into two theoretical interpretations (Wang & Li, 2014). One theory comes from the perspective of information processing, emphasizing the impact of the level of conscious involvement during the information processing on the enactment effect (Wang & Li, 2014). This includes theories such as the non-strategic processing theory (Cohen, 1981, 1989; Schatz et al., 2011) and the episodic integration theory (Kormi-Nouri, 1995). Specifically, Cohen (1981) proposed the non-strategic encoding theory, emphasizing that SPT encoding is a non-strategic form of encoding distinct from verbal memory, functioning as an automatic process. Later, Zimmer et al. (2000) confirmed the existence of an automatic pop-out mechanism during retrieval, indicating that individuals, by performing actions during the encoding stage, can automatically retrieve motor information without active search (see also Li et al., 2019). This perspective provides some support for the non-strategic nature of action memory. On the other hand, the episodic integration theory posits that the enactment effect stems from the effective integration of verbs and nouns through enacting actions (Kormi-Nouri, 1995). Enacting actions triggers a high level of self-involvement, causing individuals to focus only on the process and object of the action. This closely links the action (e.g., “wipe”) with the object (e.g., “the blackboard”), thereby more deeply connecting the semantic features of verbs and nouns during encoding and forming a more complete and coherent episodic representation (Kormi-Nouri, 1995). Such integration enhances the depth and stability of memory traces (Hainselin et al., 2014; Kormi-Nouri, 1995; Mangels & Heinberg, 2006; Senkfor et al., 2002). Behavioral evidence in support of this view shows that when the semantic processing of verbs or nouns is disrupted during encoding, the enactment effect disappears (Yu, 2017). This indicates that semantic integration is crucial for the emergence of the enactment effect. Recently, Wang et al. (2022) manipulated the degree of semantic integration of phrases (e.g., “blow a balloon” vs. “sew a toothpick”) and combined it with event-related potentials (ERPs). The study demonstrated that only under the SPT condition did phrases with poor semantic integration elicit a larger N400 component, which is considered to be closely related to semantic integration (Brown & Hagoort, 1993; Friederici, 2002), and memory performance was improved only when semantic integration was good. These results collectively suggest that SPT can trigger deeper memory traces, and the key lies in its specific enhancement of the semantic integration processing within phrases. Subsequently, research by X. Zhang et al. (2023) also indicates that the processing of action memory is inherently more conceptual, not solely reliant on the processing of motor information.

One category of explanation approaches the issue from the perspective of processing systems, namely, whether the SPT involves additional processing systems compared to the VT, such as the multimodal view (Bäckman & Nilsson, 1984; Leynes et al., 2006; Leynes & Phillips, 2008) and the motor encoding theory (Engelkamp & Zimmer, 1984; Masumoto et al., 2006). The multimodal theory suggests that the visual modality, the auditory modality, the tactual mode, and even the olfactory and gustatory modes might be involved in specific SPTs (Bäckman & Nilsson, 1984). Leynes et al. (2006) manipulated visual and tactile information through ERP and behavioral experiments, with results demonstrating that the activation of sensory information plays a crucial role in the enactment effect, thereby providing evidence to support the multiple codes theory (Leynes & Phillips, 2008). Engelkamp and Zimmer (1984), based on the principle that different stimuli should be processed by distinct processing systems, emphasize that motor, verbal, and imagery encoding operate independently, each with its own dedicated processing system. Thus, there should be a separate motor encoding system dedicated to processing actions. Moreover, compared to verbal and imagery encoding, motor encoding enhances item-specific processing, which is the key mechanism underlying the enactment effect (Zimmer & Engelkamp, 1985). Masumoto et al. (2006) conducted a study using magnetoencephalography (MEG) and found that during the early stage of a recognition test, the primary motor cortex was activated in the enactment encoding group but showed almost no activation in the verbal encoding group. In the later stage of the recognition test, the enactment group exhibited stronger activation in the right parietal region compared to the verbal repetition group. Moreover, the behavioral results indicate that the memory effect in the enactment encoding group is better than that in the verbal encoding group. This suggests that the enactment effect may be related to the activation of the motor encoding system.

### 1.2. Motor Information Representation vs. Motor Information Activation

As research progressed further, there remained an ongoing debate regarding how motor information is represented and processed. Some studies propose that motor information and verbal information may be integrated into a common conceptual or embodied representation. Both types of information are grounded in sensorimotor experiences and processed within a shared memory system, such as the Act-in (Activation-Integration) model (Versace et al., 2014). This view is consistent with embodied and grounded cognition theories, emphasizing the role of the body and sensory experiences in shaping cognitive representations (Barsalou, 2008). Some studies have proposed the perspective of motor representation (Kormi-Nouri, 1995; Russ et al., 2003), suggesting that motor encoding does not fundamentally differ from other cognitive encoding modalities. These studies emphasize that self-involvement during the process of enactment, as well as the processes of preparation, planning, and coordination of action concepts, are the key factors contributing to the enactment effect. This view holds that the enactment effect stems from a higher-order, organized cognitive representation of actions. This representation integrates the intention, goal, and semantic information of the actions, rather than merely the low-order information of the actions themselves (Kormi-Nouri, 1995; Russ et al., 2003; Ferretti, 2016). Recently, Sivashankar and Fernandes (2022) found that performing meaningful actions during encoding significantly enhanced memory performance compared to performing meaningless actions. Furthermore, it was only when performing meaningful actions that participants underwent beneficial preparatory and planning processes for the phrases during the encoding stage, thereby supporting the view of motor representation.

Some studies suggest that the activation of motor information is crucial for generating the enactment effect, proposing the concept of motor information reactivation (Macedonia & Mueller, 2016; Krönke et al., 2013; Ianì et al., 2019). This view suggests that after motor information (including factors such as the form, magnitude, and frequency of actions) is encoded, it is reactivated during the retrieval stage, thereby enhancing memory performance (Sakai, 2003). In an early functional magnetic resonance imaging (fMRI) study conducted by Nyberg et al. (2001), they directly compared brain activity during the encoding and retrieval stages under the SPT and VT conditions. The study demonstrated that different types of information activated during the encoding stage were reactivated during the retrieval stage. Moreover, compared to the VT condition, the SPT condition specifically activated the left ventral motor cortex and the left inferior parietal lobule. Liu and Wang (2018) found that this SPT effect disappeared when low-association learning materials were used, which indicates that the association between motor information and verbal information enables these two independent types of information to mutually activate each other during the retrieval stage, thereby leading to an increase in the amount of information (remembering one type of information simultaneously facilitates the retention of the other), thus enhancing memory performance. This finding provides some support for the notion that motor information reactivation can improve memory effects. Moreover, mere verbal repetition does not improve memory performance under the SPT condition (X. Zhang & Zuber, 2020).

### 1.3. Design of This Study and Research Hypotheses

Research on object memory indicates that, compared to non-manipulable objects, viewing manipulable objects or reading the names of manipulable objects can activate motor regions in the human brain (Creem-Regehr & Lee, 2005; Gerlach et al., 2002; Carota et al., 2012; Rueschemeyer et al., 2010). This indicates that the manipulability of an object affects the degree of activation of motor information. However, it remains unexplored whether performing actions on these objects—regardless of their manipulability—can enhance motor information activation and thus influence long-term memory. Therefore, this study aims to investigate the impact of the degree of enactability of action phrases on the enactment effect.

Brouillet et al. (2021) used verbs that were distant from the body (e.g., “push”) and verbs that were close to the body (e.g., “pull”) as experimental materials, asking participants to recognize or recall the verbs during the retrieval stage by enacting specific actions (e.g., “press a keyboard”, “push or pull a lever”). The study showed that enacting actions during the retrieval stage affected memory performance, and compared with neutral actions (e.g., “press a keyboard”), the actions that were closely related to verbs (“push the lever” is related to verbs that are distant from the body like “push”; “pull the lever” is related to verbs that are close to the body like “pull”) could enhance the recognition and recall of verbs. This indicates that enacting actions during the retrieval stage alone can also produce a memory effect based on actions. However, the aforementioned study did not set up a control group (no enactment group) during the retrieval stage. So, the study cannot fully explain whether there is activation of motor information or whether it is during the encoding or retrieval stages that the enactment effect is observed.

Therefore, to further investigate the role of the degree of motor information on the enactment effect, Experiment 2 incorporated enactment retrieval during the retrieval stage, requiring some participants to perform relevant actions while simultaneously verbalizing the phrases during retrieval. As mentioned above, if the degree of motor information during the retrieval stage is crucial for the enactment effect (Russ et al., 2003), then, given that substantial motor information can be activated under the combined conditions of the SPT and the high-enactability phrases, the enactment effect should be observed regardless of whether enactment retrieval is employed. Conversely, for the low-enactability-phrases condition, since relatively little motor information is activated under the SPT condition, additional activation of motor information through enactment retrieval is necessary to enhance memory performance. Therefore, the enactment effect should be observed only under the enactment retrieval, rather than under the verbal retrieval.

Based on this, the present study designed two experiments to explore the influence of the degree of enactability on the enactment effect from a combined perspective of encoding and retrieval. Specifically, Experiment 1 adopted the SPT paradigm and utilized verb–noun phrases with varying degrees of enactment as experimental materials to investigate, from the encoding stage, how different degrees of enactment (high-enactability phrases, e.g., “twist a bottle cap”, “toss a coin”; low-enactability phrases, e.g., “sprout a bud”, “pass a level”) affect the enactment effect. It is posited that the actions denoted by entire high-enactability phrases (i.e., the entire verb–noun phrase) are relatively concrete and well-defined, and that these phrases entail richer motor information; therefore, under SPT conditions, they produce more distinct memory traces and are more likely to yield an enactment effect. In contrast, low-enactability phrases contain relatively sparse motor information, resulting in less distinct memory traces under enactment conditions and making it more difficult for an enactment effect to emerge. Consequently, Experiment 1 hypothesizes that an enactment effect will be observed for the high-enactability-phrases condition but not for the low-enactability-phrases condition. Building upon Experiment 1, Experiment 2 further explores the influence of the degree of enactability on the enactment effect from the perspective of retrieval.

## 2. Experiment 1: Exploring the Influence of the Degree of Enactability on the Enactment Effect During the Encoding Stage

### 2.1. Method

#### 2.1.1. Design

A 2 (encoding condition: SPT or VT, between-subjects) × 2 (degree of enactability: high or low, within-subjects) mixed design was used. The dependent variable was the proportion of items that were correctly recalled. It is noteworthy that the encoding conditions were designed as a between-subjects design to minimize interference from strategy transfer, order effects, and fatigue during the encoding process (Engelkamp & Zimmer, 1994; Li et al., 2017). Although a between-subjects design may be statistically less powerful than a within-subjects design (Roberts et al., 2022), the choice in this study prioritized ensuring the independence and experimental validity of each encoding condition and aligns with common practices in this field (Cohen, 1981; Sivashankar & Fernandes, 2022).

#### 2.1.2. Participants

Before the experiment, we used G*Power 3.1 (Faul et al., 2007) to calculate the sample size. Drawing on the previous research (Sivashankar & Fernandes, 2022), the parameters were set as α = 0.05, 1 − β = 0.80, and *f* = 0.25, yielding a minimum sample size of 34. Ultimately, we recruited 40 participants (including 8 males) from Jiangsu Normal University in China, with ages ranging from 18 to 26 years (*M* = 21.55, *SD* = 2.06). All participants were right-handed, had normal or corrected-to-normal vision, had no history of psychiatric disorders, and had never participated in similar experiments before. This study was approved by the Ethics Committee of Jiangsu Normal University, and written informed consent was obtained from all participants.

#### 2.1.3. Materials

Initially, we selected 50 action phrases from a common dictionary of modern Chinese as research samples. Before the formal experiment, a word-rating test was conducted by recruiting 18 students (who did not participate in the formal experiment), who were asked to rate the familiarity and degree of enactability of the phrases on a seven-point scale. For the rating of the phrases’ degree of enactability, participants were instructed to assess the action referred to by the entire verb–noun phrase and judge whether it was easy to use their right hand to perform a concrete physical action to represent the phrase. They were informed that higher ratings indicate that the action described by the phrase is easier to perform. Subsequently, we screened out 38 action phrases that exhibited no significant differences in familiarity. Finally, following the method of Liu and Wang (2018), phrases with a score distribution above 73% (an average grade greater ≥5.11 points) were defined as high-enactability phrases, and those below 27% (an average grade greater ≤1.89 points) were defined as low-enactability phrases, ensuring that the two groups were adequately separated along the operability dimension. The data were analyzed using an independent-samples *t*-test. The results showed that there was a significant difference in the degree of enactability between the high-enactability (6.32 ± 0.30) and the low-enactability (1.46 ± 0.20) phrases, *t* (36) = 58.85, *p* < 0.001, Cohen’s *d* = 19.06. No significant difference was observed in familiarity between the high-enactability (6.87 ± 0.07) and low-enactability (6.83 ± 0.08) phrases, *t* (36) = 1.64, *p* = 0.11, Cohen’s *d* = 0.53.

The experimental materials consisted of a word list comprising 38 verb–noun phrases, including 19 verb–noun phrases with a high degree of enactability and 19 verb–noun phrases with a low degree of enactability. Each phrase was composed of 2 to 3 Chinese characters, and neither the verbs nor the nouns within the phrases contained obscure or uncommon words. High-enactability phrases referred to common object-directed actions with clear action representations, allowing participants to generate nearly identical simulated actions (e.g., “twist a bottle cap” or “toss a coin”). In contrast, low-enactability phrases were not unperformable; rather, they required participants to mobilize richer cognitive resources to transform abstract concepts into highly individualized symbolic gestures (e.g., forming a “V” shape with the hand to represent “sprout a bud”, or making a fist with one hand and pulling it downward to represent “pass a level”, a gesture interpreted as a celebratory action signifying successful completion). Therefore, all phrases used in the experiment could be assigned a meaningful, non-arbitrary enacted performance, ensuring that the comparisons were made along a continuum of intensity within the same cognitive process, rather than between tasks of different natures. The full list of experimental phrases is provided in Appendix A to facilitate replication.

#### 2.1.4. Procedure

The experimental procedure in this study followed that of Li et al. (2017). Experiment 1 was divided into three stages: learning, interference, and retrieval.

During the learning stage, a black fixation cross (“+”) was presented at the center of the computer screen for 1000 ms, followed by the presentation of each phrase for 6 s in random order. A total of 40 participants were randomly assigned to either the SPT condition or the VT condition, with 20 participants in each group. In the SPT condition, participants were required to silently read each phrase while simultaneously physically enacting the corresponding action with their right hand, represented by the phrase. In the VT condition, participants were only required to silently read the presented phrases without enacting any corresponding actions. After the learning stage, the interference phase began. Three puzzle-style math problems were simultaneously presented on the computer screen, and participants were required to answer them within 3 min. After the interference task, a prompt appeared on the screen instructing the participant to recall the learned action phrases. The participant was required to write down the action phrases they just learned within 5 min.

#### 2.1.5. Scoring

Given that this study employed a recall test, participants might make spelling errors or write phrases that were semantically similar to the learning materials during the response process, introducing subjectivity into the scoring of memory performance. Therefore, this study adopted a strict scoring criterion, meaning that only completely correct phrases were included in the final analysis. The recall score is calculated based on accuracy.

#### 2.1.6. Data Analysis

The primary dependent variable in this study was the recall accuracy rate. The data analysis aimed to systematically examine the influence of the degree of enactability on memory performance and their interaction. To this end, using SPSS 23.0, recall accuracy was analyzed with a 2 (encoding condition: SPT vs. VT; between-subjects) × 2 (degree of enactability: high vs. low; within-subjects) mixed-design ANOVA (i.e., split-plot ANOVA). Simple effects analyses were performed using the Bonferroni correction.

### 2.2. Results

**Figure 1:**
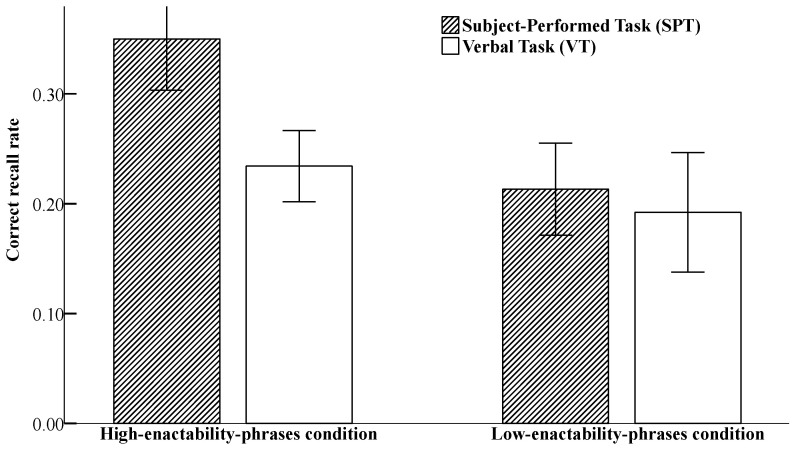
The bar chart depicts recall accuracy rates under different conditions. The error bars represent standard errors of the mean.

The descriptive statistical data of recall accuracy rates across different encoding conditions and degrees of enactment are presented in Figure 1.

A 2 (encoding condition: SPT vs. VT; between-subjects) × 2 (degree of enactability: high vs. low; within-subjects) mixed-design ANOVA was conducted to analyze recall accuracy. An alpha level was set at ≤0.05. Our results revealed a significant main effect of encoding condition, *F* (1, 38) = 7.78, *p* = 0.008, *η*_p_^2^ = 0.17, 95% CI = [0.02, 0.12], suggesting that the recall accuracy rate under the SPT condition (*M* = 0.29, *SE* = 0.02) was significantly higher than that under the VT condition (*M* = 0.21, *SE* = 0.02). The main effect of degree of enactability was significant, *F* (1, 38) = 26.15, *p* < 0.001, *η*_p_^2^ = 0.41, 95% CI = [0.05, 0.13], showing that the recall accuracy rate for the high-enactability-phrases condition (*M* = 0.29, *SE* = 0.01) was significantly higher than that for the low-enactability-phrases condition (*M* = 0.20, *SE* = 0.02).

The interaction between encoding condition and degree of enactability was significant, *F* (1, 38) = 7.33, *p* = 0.01, *η*_p_^2^ = 0.16. Furthermore, simple effect analyses of this interaction revealed that for the high-enactability-phrases condition, the recall accuracy rate under the SPT condition (*M* = 0.35, *SE* = 0.02) was significantly higher than that under the VT condition (*M* = 0.23, *SE* = 0.02), *F* (1, 38) = 18.12, *p* < 0.001, *η*_p_^2^ = 0.32, 95% CI = [0.06, 0.17]; however, for the low-enactability-phrases condition, no significant difference in recall accuracy rates was observed between the SPT (M = 0.21, SE = 0.02) and VT conditions (M = 0.19, SE = 0.02), *F* (1, 38) = 0.41, *p* = 0.53, *η*_p_^2^ = 0.01, 95% CI = [−0.09, 0.05].

### 2.3. Discussion

The results of Experiment 1 demonstrated that for the high-enactability-phrases condition, the recall performance under the SPT condition was significantly higher than that under the VT condition; however, for the low-enactability-phrases condition, no significant difference in recall performance was observed between the SPT and VT conditions. This suggests that an enactment effect was observed for the high-enactability-phrases condition but not for the low-enactability-phrases condition. These findings align with the hypotheses, indicating that the degree of enactability influences the enactment effect.

These results support the findings of Sivashankar and Fernandes (2022), which suggest that enacting meaningful actions, as opposed to irrelevant motor gestures, enhances memory performance. In other words, when actions are semantically related to the phrases, memory performance is further improved. According to the study by Sivashankar and Fernandes (2022), the meaningless actions they employed were either freely chosen actions deemed unrelated to the target phrases (Experiment 1) (participants were free to select and perform any action they deemed was unrelated to the word) or pretending to write the target word in the air (Experiment 2). The research suggests that the core of meaningless actions lies in the low referential matching between the action and the phrase. Therefore, in this study, participants were required to perform semantically related actions for both high-enactability and low-enactability phrases, with the key difference being whether the performed action could easily and accurately match the phrase. High-enactability phrases describe actions that are common and familiar to participants in daily life. Thus, under the SPT condition, they were able to accurately enact these actions. This accurate embodied enactment likely enhanced the semantic integration between action simulation and the verbal representation of the phrases, thereby activating richer and more effective motor information. In contrast, low-enactability phrases describe vague actions. Although participants attempted to use actions that they felt could refer to the phrases, they found it difficult to perform action simulations that closely matched the meaning of the phrases. Consequently, the degree of semantic integration during encoding was lower, and the activated motor information was weaker. In other words, the actions performed by individuals for the phrases were more aligned with the essential connotations of the phrases, thus activating more abundant and effective motor information. However, when participants were faced with low-enactability phrases, they were unclear about how to perform the actions represented by these phrases. As a result, the actions they performed may not have matched the actions denoted by the phrases well, leading to the activation of less effective motor information.

Experiment 1 explored the impact of the degree of enactability contained in the phrases on the enactment effect from the perspective of encoding. However, in Experiment 1, no enactment effect was observed for the low-enactability-phrases condition, which might have been due to insufficient activation of motor information. Therefore, to further test this hypothesis, Experiment 2 introduced enactment during the retrieval stage, aiming to achieve a certain degree of activation of motor information so that an enactment effect would be observed.

## 3. Experiment 2: Exploring the Influence of the Degree of Enactability on the Enactment Effect During the Retrieval Stage

The results of Experiment 1 indicated that the enactment effect was observed only for the high-enactability-phrases condition, while it was not observed for the low-enactability-phrases condition. This pattern might be due to insufficient activation of motor information during the encoding stage for the latter. To test this hypothesis, Experiment 2 introduced an enactment retrieval task during the retrieval stage, aiming to examine whether the enactment effect could be elicited for the low-enactability-phrases condition. The study posits that for the low-enactability-phrases condition, the activation of motor information during encoding is limited and insufficient to produce an enactment effect; however, if this is combined with the reactivation of motor information during retrieval, the activation of motor information may reach a certain threshold, allowing the enactment effect to emerge. Therefore, the research hypothesizes that for the high-enactability-phrases condition, the enactment effect will be observed in both enactment retrieval and verbal retrieval conditions; for low-enactability-phrases conditions, the enactment effect will be observed only under the combined condition of SPT and enactment retrieval, but not under verbal retrieval.

### 3.1. Method

#### 3.1.1. Design

A 2 (encoding condition: SPT vs. VT; between-subjects) × 2 (retrieval condition: enactment vs. verbal; between-subjects) × 2 (degree of enactability: high vs. low; within-subjects) mixed design was used. The dependent variable was the proportion of correctly recalled items.

#### 3.1.2. Participants

Before the experiment, we used G*Power 3.1 (Faul et al., 2007) to calculate the sample size. Drawing on previous research (Sivashankar & Fernandes, 2022), the parameters were set as α = 0.05, 1 − β = 0.80, and *f* = 0.25, yielding a minimum sample size of 48. Ultimately, 80 participants (including 19 males) were recruited from a university, with ages ranging from 18 to 26 years (*M* = 21.21, *SD* = 1.81). All participants were right-handed, had normal or corrected-to-normal vision, had no history of psychiatric disorders, and had never participated in similar experiments before. This study was approved by the Ethics Committee of Jiangsu Normal University, and written informed consent was obtained from all participants.

#### 3.1.3. Materials

The experimental materials were the same as those used in Experiment 1.

#### 3.1.4. Procedure

The encoding and interference stages were the same as in Experiment 1, but the retrieval stage differed from that of Experiment 1. Under the verbal retrieval condition, participants verbally recalled the phrases they had learned without performing any actions. Under the enactment retrieval condition, participants enacted actions related to the phrases with their right hand while verbally recalling the phrases.

Notably, unlike Experiment 1, which used written recall, the recall test in Experiment 2 adopted an oral recall format. This design modification was implemented to avoid potential coordination interference between the required enactment and the act of writing during the recall phase. Under the enactment retrieval condition in Experiment 2, requiring participants to write while performing the specified actions could introduce additional cognitive and motor loads; therefore, a simpler and more feasible oral recall method was employed. We have discussed the potential impact of this methodological difference on the comparability of results in the Discussion section.

#### 3.1.5. Scoring

The scoring method was the same as that in Experiment 1.

#### 3.1.6. Data Analysis

Using SPSS 23.0, recall accuracy was analyzed with a 2 (encoding condition: SPT vs. VT; between-subjects) × 2 (retrieval condition: enactment retrieval vs. verbal retrieval; between-subjects) × 2 (degree of enactability: high vs. low; within-subjects) mixed-design ANOVA. Since the enactment effect (the recall advantage of SPT over VT) is not a fixed pattern, its occurrence depends simultaneously on the degree of enactability and the retrieval method. This suggests that the activation and utilization of motor information is a dynamic process, possibly involving the summation of activation levels during the encoding and retrieval stages. To parse this complex interaction, we subsequently performed a series of simple effect analyses: The interaction between encoding condition and degree of enactability was analyzed separately under different retrieval conditions. To compare processing differences between action memory and working memory, we also analyzed the interaction between the degree of enactability and the retrieval condition separately under different encoding conditions. Finally, we calculated the enactment effect size (SPT performance—VT performance) and used independent-samples *t*-tests to compare the magnitudes of the enactment effect between enactment retrieval and verbal retrieval conditions for different degrees of enactment.

### 3.2. Results

**Figure 2:**
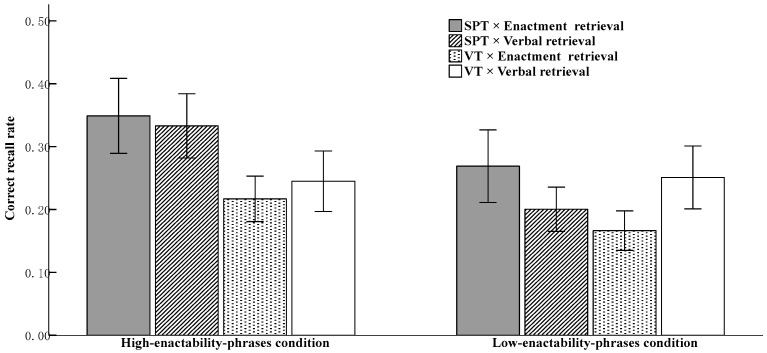
The bar chart depicts recall accuracy rates under different conditions. The error bars represent standard errors of the mean. Notes: As shown in Figure 2, when encoding occurred under the VT condition, recall appeared numerically higher under verbal retrieval than under enactment retrieval, particularly for the low-enactability phrases. This pattern may appear counterintuitive. A possible explanation involving an encoding–retrieval mismatch and the additional verbal–action translation and coordination demands introduced by enactment at retrieval is discussed in the General Discussion (Section 4.2).

The descriptive statistical data on recall accuracy rates under different conditions are presented in Figure 2.

A 2 (encoding condition: SPT vs. VT; between-subjects) × 2 (retrieval condition: enactment vs. verbal; between-subjects) × 2 (degree of enactability: high vs. low; within-subjects) mixed-design ANOVA was conducted to analyze recall accuracy. An alpha level was set at ≤0.05. The results revealed a significant main effect of encoding condition, *F* (1, 76) = 12.70, *p* = 0.001, *η*_p_^2^ = 0.14, 95% CI = [0.03, 0.11], suggesting that the recall accuracy rate under the SPT condition (*M* = 0.29, *SE* = 0.01) was significantly higher than that under the VT condition (M = 0.22, SE = 0.01) Additionally, the main effect of degree of enactability was significant, *F* (1, 76) = 28.41, *p* < 0.001, *η*_p_^2^ = 0.27, 95% CI = [0.04, 0.09], showing that the recall accuracy rate for the high-enactability-phrases condition (*M* = 0.29, *SE* = 0.01) was significantly higher than that for the low-enactability-phrases condition (*M* = 0.22, *SE* = 0.01). The main effect of retrieval condition was not significant, *F* (1, 76) = 0.14, *p* = 0.72, *η*_p_^2^ = 0.002, 95% CI [−0.05, 0.03], showing that there was no significant difference in recall accuracy between the enactment retrieval condition (*M* = 0.25, *SE* = 0.01) and the verbal retrieval condition (*M* = 0.26, *SE* = 0.01).

A significant three-way interaction was found among encoding condition, retrieval condition, and degree of enactability, *F* (1, 76) = 5.11, *p* < 0.03, *η*_p_^2^ = 0.06.

To clarify how the retrieval conditions modulate the enactment effect, we further examined the interaction between encoding condition and degree of enactability separately under different retrieval conditions. Looking at the enactment retrieval separately: the main effect of the degree of enactability was significant, *F* (1, 38) = 15.06, *p* < 0.001, *η*_p_^2^ = 0.28, 95% CI = [0.03, 0.10], showing that the recall accuracy for the high-enactability-phrases condition (*M* = 0.28, *SE* = 0.02) was significantly greater than that for the low-enactability-phrases condition (*M* = 0.22, *SE* = 0.02); the main effect of the encoding condition was also significant, *F* (1, 38) = 17.922, *p* < 0.001, *η*_p_^2^ = 0.32, 95% CI = [0.06, 0.17], showing that the recall accuracy under the SPT condition (*M* = 0.31, *SE* = 0.02) was significantly higher than that under the VT condition (*M* = 0.19, *SE* = 0.02), indicating that the enactment effect was observed for both high-enactability and low-enactability-phrases conditions; the interaction between the degree of enactability and the encoding condition was not significant, *F* (1, 76) = 0.77, *p* = 0.39, *η*_p_^2^ = 0.02.

Looking at verbal retrieval separately: The main effect of degree of enactability was significant, *F* (1, 38) = 13.40, *p* = 0.001, *η*_p_^2^ = 0.26, 95% CI [0.03, 0.10], showing that the recall accuracy for the high-enactability-phrases condition (*M* = 0.29, *SE* = 0.02) was significantly greater than that for the low-enactability-phrases condition (*M* = 0.23, *SE* = 0.02); the main effect of encoding condition was not significant, *F* (1, 38) = 0.51, *p* = 0.48, *η*_p_^2^ = 0.01, 95% CI [−0.03, 0.07], showing that there was no significant difference in recall accuracy between the SPT condition (*M* = 0.27, *SE* = 0.02) and the VT condition (*M* = 0.25, *SE* = 0.02); the interaction effect between degree of enactability and encoding condition was significant, *F*(1, 38) = 16.06, *p* < 0.001, *η*_p_^2^ = 0.30. Simple effect analysis revealed that for the high-enactability-phrases condition, recall accuracy under the SPT condition (*M* = 0.33, *SE* = 0.02) was significantly higher than that under the VT condition (*M* = 0.25, *SE* = 0.02), *F*(1, 38) = 6.93, *p* = 0.01, *η*_p_^2^ = 0.15, 95% CI [0.02, 0.16]. For the low-enactability-phrases condition, there was no significant difference in recall accuracy between the SPT condition (*M* = 0.20, *SE* = 0.02) and the VT condition (*M* = 0.25, *SE* = 0.02), *F* (1, 38) = 2.99, *p* = 0.09, *η*_p_^2^ = 0.07, 95% CI [−0.11, 0.01].

To further examine how retrieval conditions interact with the degree of enactability under different encoding conditions, additional analyses were conducted. The interaction between retrieval condition and degree of enactability was analyzed separately under each encoding condition. This approach also facilitates comparison with findings on the enactment effect in working memory. Looking at the high-enactability-phrases condition separately: The main effect of encoding condition was significant, *F* (1, 76) = 21.65, *p* < 0.001, *η*_p_^2^ = 0.22, 95% CI [−0.06, 0.16], showing that recall accuracy under the SPT condition (*M* = 0.34, *SE* = 0.02) was significantly higher than under the VT condition (*M* = 0.23, *SE* = 0.02), indicating that an enactment effect was observed under both retrieval conditions; the main effect of retrieval condition was not significant, *F* (1, 76) = 0.06, *p* = 0.80, *η*_p_^2^ = 0.001, 95% CI [−0.05, 0.04]. There was no significant difference in recall accuracy between the enactment retrieval condition (*M* = 0.28, *SE* = 0.02) and the verbal retrieval condition (*M* = 0.29, *SE* = 0.02); the interaction between retrieval condition and encoding condition was not significant, *F* (1, 76) = 0.87, *p* = 0.36, *η*_p_^2^ = 0.01.

Looking at the low-enactability-phrases condition separately: The main effect of encoding condition was not significant, *F* (1, 76) = 1.47, *p* = 0.23, *η*_p_^2^ = 0.02, 95% CI [−0.02, 0.07], showing that there was no significant difference in recall accuracy between the SPT condition (*M* = 0.24, *SE* = 0.02) and the VT condition (*M* = 0.21, *SE* = 0.02). The main effect of retrieval condition was not significant, *F* (1, 76) = 0.14, *p* = 0.71, *η*_p_^2^ = 0.002, 95% CI [−0.05, 0.04], showing that there was no significant difference in recall accuracy between the enactment retrieval condition (*M* = 0.22, *SE* = 0.02) and the verbal retrieval condition (*M* = 0.23, *SE* = 0.02). However, the interaction between retrieval condition and encoding condition was significant, *F* (1, 76) = 12.72, *p* = 0.001, *η*_p_^2^ = 0.14. Simple effect analysis showed that under the SPT condition, recall accuracy under the enactment retrieval condition (*M* = 0.27, *SE* = 0.02) was significantly higher than under the verbal retrieval condition (*M* = 0.20, *SE* = 0.02), *F* (1, 76) = 5.10, *p* = 0.03, *η*_p_^2^ = 0.06, 95% CI [−0.01, 0.13]; under the VT condition, recall accuracy under the enactment retrieval condition (*M* = 0.17, *SE* = 0.02) was significantly lower than under the verbal retrieval condition (*M* = 0.25, *SE* = 0.02), *F* (1, 76) = 7.76, *p* = 0.007, *η*_p_^2^ = 0.10, 95% CI [−0.15, −0.02].

In further analyses, this study conducted an independent-samples *t*-test on the magnitude of the enactment effect (enactment effect = recall performance under the SPT condition − recall performance under the VT condition) under enactment retrieval and verbal retrieval for the high-enactability-phrases condition. The results indicated that for the high-enactability-phrases condition, there was no significant difference in the enactment effect between the enactment retrieval (*M* = 0.13, *SE* = 0.14) and verbal retrieval conditions (*M* = 0.13, *SE* = 0.14), *t* (38) = 0.89, *p* = 0.38, Cohen’s *d* = 0.28, 95% CI = [−0.06, 0.14]. For the low-enactability-phrases condition, there was a significant difference in the enactment effect between enactment retrieval (*M* = 0.10, *SE* = 0.14) and verbal retrieval (*M* = −0.05, *SE* = 0.13), *t* (38) = 3.63, *p* = 0.001, Cohen’s *d* = 1.15, 95% CI = [0.07, 0.24].

### 3.3. Discussion

The results of Experiment 2 demonstrated that, under the verbal retrieval condition, the enactment effect was observed for the high-enactability phrases but not for the low-enactability phrases, replicating the results of Experiment 1. However, under the enactment retrieval condition, the enactment effect was observed for the low-enactability phrases, validating the research hypothesis. This indicates that enactment during both encoding and retrieval stages enhances the activation of motor information. Meanwhile, situations where no enactment occurs during encoding but enactment is carried out during retrieval may interfere with memory performance.

## 4. General Discussion

### 4.1. Exploring the Influence of the Degree of Enactability on the Enactment Effect During the Encoding Stage

The results of Experiment 1 in this study indicate that an enactment effect was observed for the high-enactability-phrases condition but not for the low-enactability-phrases condition. Consistent with our hypothesis, the findings from Experiment 1 indicate that the degree of enactability influences the enactment effect. In this study, high-enactability phrases are associated with better memory performance, which may be attributed to more effective activation of motor information during encoding, compared with low-enactability phrases. That is, the actions enacted by individuals match the essential connotations of the phrases more closely, leading to the activation of more abundant and effective motor information. Conversely, low-enactability phrases result in a lower degree of match between the actions participants enact to represent the phrases and the actions the phrases actually denote, thus activating less effective motor information.

Moreover, motor information plays a crucial role in the generation of the enactment effect (Heil et al., 1999). The theory of motor information reactivation posits that once motor information (including factors such as the form, magnitude, and frequency of movements) is encoded, it will be reactivated during the retrieval stage, thereby enhancing memory performance (Sakai, 2003). This view aligns with the broader sensorimotor simulation theory (Barsalou, 2008), which emphasizes that recalling perceptual information reactivates the neural networks responsible for initially processing that information during encoding, suggesting that retrieval is accompanied by the reactivation of relevant brain regions (Slotnick, 2004). Notably, the Act-In model proposed by Versace et al. (2014) posits that memory traces encompass multi-component experiences, including sensory, motor, and affective elements. The emergence of knowledge relies on the activation and integration processes among these memory traces. Within this model, action manipulation can facilitate strong integration among the components within a trace (e.g., verbs, nouns, action features, contextual information), thereby enhancing the specificity and retrievability of the trace.

However, the activation of motor information is not an “all-or-none” process; its intensity may vary with the nature of the task and materials (Schaller et al., 2017; Tian et al., 2020). For instance, Tian et al. (2020) used verb–noun combinations to examine the involvement of the motor system during the processing of action phrases with literal meanings (e.g., “catch the ball”), metaphorical meanings (e.g., “catch the meaning”), and abstract meanings (e.g., “understand the meaning”). The results indicated a gradient attenuation of motor activation from literal to metaphorical to abstract meanings, suggesting that the motor system participates in language processing in a gradual, gradient manner. These findings imply that the degree of enactability of phrases may directly influence the activation strength of motor information during the encoding stage: low-enactability phrases, due to their difficulty in precisely matching specific actions, may elicit weaker activation of motor information, whereas high-enactability phrases can trigger stronger and more effective encoding of motor information.

Therefore, in this study, compared to enacting phrases with low-enactability during the encoding stage, enacting phrases with high-enactability can activate more motor information, and potentially lead to the reactivation of a greater amount of motor information during the retrieval stage. This results in the emergence of the enactment effect for the high-enactability-phrases condition, while no such effect is observed for the low-enactability-phrases condition. Consequently, the findings of this study support the viewpoint of motor information reactivation (Nyberg et al., 2001; Ianì et al., 2019; Sakai, 2003; Liu & Wang, 2018).

Further interpretation of the research results: when participants perform corresponding actions based on the meanings of phrases, motor information is activated and will be reactivated during the retrieval stage; compared with high-enactability phrases, the motor information contained in low-enactability phrases is so scarce that the enactment effect cannot be observed. This indicates that the quantity of motor information is crucial for the emergence of the enactment effect. Therefore, to a certain extent, the results of this study also expand the theory of motor information reactivation.

Undeniably, participants can only enact actions after achieving a sufficient conceptual understanding of the motor phrases (Kormi-Nouri, 1995; Knopf et al., 2005; Wang et al., 2022; Sivashankar & Fernandes, 2022; X. Zhang et al., 2023). For this very reason, when participants enact actions corresponding to phrases with high enactability or low enactability, these actions maintain semantic relevance to the performed phrases. However, in this study, phrases with low enactability resulted in a lower degree of match between the actions performed by participants and the phrases themselves. Therefore, no enactment effect occurred.

### 4.2. Exploring the Influence of the Degree of Enactability on the Enactment Effect During the Retrieval Stage

The results of Experiment 2 demonstrate that, under the condition of verbal retrieval, an encoding enactment effect was observed for the high-enactability-phrases condition but not for the low-enactability-phrases condition. This finding replicates the results of Experiment 1, further confirming that the degree of enactability is crucial for the emergence of the enactment effect. Although the enactment effect was statistically observed in both experiments for high-enactability phrases and verbal retrieval conditions, in Experiment 2, there was a notable decline in the effect size of the SPT under verbal retrieval and high-enactability-phrases conditions compared to that in Experiment 1, which might lead some to question the reliability of the current findings. A possible reason is that, compared to Experiment 1, Experiment 2 replaced the written recall task with an oral recall task. Janczyk et al. (2018) demonstrated that there are differences in recall performance between written and oral recall tasks when dealing with item sets of a certain size, and that written recall may exhibit higher accuracy compared to oral recall (J. Zhang et al., 2021). Therefore, this discrepancy might have influenced the consistency of the results between Experiment 2 and Experiment 1 in the present study. Nevertheless, the findings from Experiment 1 were still corroborated in Experiment 2.

Under the enactment retrieval, enactment effects were observed for both the high-enactability phrases and the low-enactability-phrases conditions. This result aligns with the encoding specificity principle (Tulving & Thomson, 1973), which posits that memory performance is superior when the encoding and retrieval conditions are congruent compared to when they are incongruent. Therefore, when the encoding condition is SPT and the retrieval condition is enactment retrieval, memory performance is better than when the encoding condition is VT and the retrieval condition is enactment retrieval. Consequently, regardless of the degree of enactability, an encoding enactment effect is observed under the enactment retrieval condition. Notably, a mismatch occurred between encoding and retrieval conditions in this study. Specifically, encoding involved VT, while retrieval required enactment. This inconsistency does not align with the encoding specificity principle and may therefore impair memory retrieval performance. Particularly, for the low-enactability-phrases condition, the enhancing effect of enactment retrieval on memory was weaker, and the matching degree between actions and phrases was weaker compared to the high-enactability phrases during enactment retrieval. In other words, enactment retrieval for the low-enactability-phrases condition tended to consume more cognitive resources from participants (Eggert et al., 2022), leading to interference. Consequently, in this study, situations wherein no enactment occurs during encoding but enactment is carried out during retrieval may interfere with memory performance.

However, studies in the field of working memory have shown that memory performance under the VT condition for action recall is significantly better than that for verbal recall (Allen et al., 2020; Allen & Waterman, 2015; Jaroslawska et al., 2016, 2018; Waterman et al., 2017; Yang et al., 2015). Jaroslawska et al. (2018) employed three dual-task paradigms to explore the reasons behind this phenomenon: articulatory suppression (continuously saying “1, 2, 3”), backward counting (continuously subtracting 3 from a given three-digit number), and motor suppression (performing a series of brief, repetitive fine or gross movements with the dominant hand). The results indicated that the advantage of action recall over verbal recall persisted under articulatory suppression and backward counting interference conditions, but gross motor movements selectively interfered with the advantage of action recall. This suggests the existence of a specialized cognitive system for temporarily maintaining spatial–motor representations (Smyth & Pendleton, 1989, 1990). The advantage in action recall relies on a limited-capacity store used to temporarily retain the motor, spatial, and temporal features of intended actions (Jaroslawska et al., 2018). Subsequently, Li et al. (2022) proposed that there is an “action output” buffer in the working memory system for processing action commands. This implies that in working memory tasks, participants generate representations of action sequences in their minds from the beginning of the task (Waterman et al., 2017; Yang et al., 2015), making subsequent action recall not a temporary conversion from words to actions but a direct retrieval of pre-existing action plans. However, in action memory, when participants silently read phrases during the encoding stage, they may not form action cues that match the items, especially with low-enactability materials. Introducing actions during the retrieval stage may increase the burden of switching between the action and verbal processing systems, leading to resource competition and interference, thereby causing the performance of actions during retrieval to disrupt recall instead.

The purpose of Experiment 2 was to investigate the impact of the activation level of motor information during retrieval on the enactment effect. The results indicated that, under the condition of verbal retrieval, the enactment effect was observed for the high-enactability condition but not for the low-enactability condition; under the condition of enactment retrieval, the enactment effect was observed both for the high-enactability phrases and the low-enactability-phrases conditions. One plausible interpretation, in line with the motor information reactivation theory, is that a cumulative effect between motor activation during encoding and reactivation during retrieval may occur. Specifically, although no enactment effect was observed under the verbal retrieval and low-enactability conditions, the enactment effect did occur when participants enacted actions again during the retrieval stage. To some extent, this indicates that during the retrieval stage, participants activated motor information through enacting actions (Brouillet et al., 2021). This information was then superimposed with the motor information activated during the encoding phase, enabling the degree of motor information activation to reach a level high enough to enhance memory.

In contrast, for the high-enactability-phrases condition, the enactment effect was observed regardless of whether enactment retrieval was involved. Therefore, a follow-up statistical comparison of the magnitude of the enactment effect (SPT − VT) indicated that, for the high-enactability-phrases condition, there was no statistically significant difference in the enactment effect between enactment retrieval and verbal retrieval, whereas for the low-enactability-phrases condition, there was a statistically significant difference between the two retrieval conditions. This suggests that, for the high-enactability phrases, this cumulative effect is not pronounced, likely because the motor information has already been fully activated during encoding, rendering the impact of reactivation during retrieval relatively limited. Specifically, for the high-enactability phrases, enacting actions during encoding activates a greater amount of motor information, providing a robust encoding effect (Li et al., 2017), so the motor information reactivated during retrieval is relatively limited. Therefore, for the high-enactability-phrases condition, regardless of whether actions are performed during the retrieval stage, an enactment effect will be observed when participants perform actions during the encoding stage.

However, the existing literature presents inconsistent findings regarding whether enactment retrieval during the retrieval stage has a memory-enhancing effect. The results of this study align with the conclusions of Brouillet et al. (2021), indicating that enactment during retrieval enhances memory. However, other research has not observed such benefits (e.g., Zeelenberg et al., 2020). One possible explanation lies in the degree of motor information activation achieved during encoding and retrieval. In studies where enactment during retrieval failed to improve memory, it may be due to either insufficient strength of the motor traces formed during encoding or a poor match between the actions executed during retrieval and those encoded, thereby failing to reach the degree of motor information activation required to facilitate memory. In the present study, for low-enactability phrases, a significant enactment effect emerged only when enactment during retrieval supplemented the weaker motor encoding, such that the total motor information activation exceeded a critical degree. Conversely, for high-enactability phrases, the motor traces formed during encoding were already robust enough, making additional enhancement during retrieval less critical (Li et al., 2017). Therefore, it is argued that the positive effect of enactment during retrieval is not universal but rather depends on the initial strength and specificity of the motor representations formed during encoding.

A deeper analysis reveals that when enactment occurs in only one stage (either the encoding stage or the retrieval stage), the enactment effect is not consistently observed across different degrees of enactment. However, when enactment is present in both the encoding and retrieval stages, enactment effects are observed under varying degrees of enactment. This suggests that, for an enactment effect to occur under different conditions, it may not only require the activation of motor information but also necessitate that the motor information be activated to a certain degree to effectively enhance memory performance.

### 4.3. Limitations and Prospects

This study investigated the influence of varying degrees of enactment on the enactment effect from a combined perspective of encoding and retrieval, providing new evidence for understanding the mechanisms underlying the enactment effect. However, several limitations remain. First, all participants were healthy young university students, and the experimental materials consisted of short Chinese action phrases, which limits the generalizability of the findings to different populations, more complex materials, or other linguistic and cultural contexts. Future research should validate these results using broader samples and materials with higher ecological validity. Second, while the study proposes that motor information activation must reach a certain threshold to enhance memory, it may lack direct neural evidence (e.g., EEG, fMRI) for activation levels during encoding and retrieval stages, and it has not entirely ruled out alternative explanations such as attentional load or task switching. More importantly, the focus of this work is to provide mechanistic evidence for the enactment effect. Broader theoretical questions regarding action memory as a whole require future investigation combining multi-level evidence, including neuroimaging, within a broader perspective (e.g., Ratner & Foley, 2020).

It is noteworthy that the activation of motor information not only plays a crucial role in memory performance during encoding and retrieval but may also be critical for the long-term consolidation process of memory traces. Research demonstrates that the sensorimotor simulations occurring during language comprehension not only influence immediate cognitive processing but also enhance memory performance in delayed memory tasks. This effect has been observed in both native (Pecher et al., 2009) and non-native language comprehenders (Zeelenberg et al., 2025). This suggests that sufficient activation of motor information not only boosts immediate memory performance but may also improve the stability of long-term memory by facilitating integration and consolidation within the memory system. Future research could further explore the sustained mechanisms of motor information in the enactment effect from the perspective of memory consolidation.

This study also provides new insights for childhood education and even the education of special populations. The results indicate that the degree of enactability influences the enactment effect. When conducting verbal instruction, teachers can utilize visualized actions to assist teaching based on the nature of different learning contents, and even encourage children to produce actions that better match the learning materials, thereby enhancing learning efficiency and flexibility. For groups with special educational needs (such as children with learning disabilities or autism spectrum disorders), this multi-sensory participatory teaching approach may help them comprehend the learning content and overcome limitations in symbolic understanding.

## 5. Conclusions

This study explored the impact of motor information activation on the enactment effect from the perspectives of encoding and retrieval. The results demonstrate that for the high-enactability-phrases condition, the activation of motor information during both encoding and retrieval stages effectively enhances memory performance. In contrast, for the low-enactability-phrases condition, enacting actions during the encoding stage alone may not be sufficient to significantly improve memory. However, when motor information is reactivated to an appropriate degree through enacting actions during the retrieval stage, memory enhancement can be achieved. Therefore, the findings validate that the degree of motor information activation during both the encoding and retrieval stages is crucial for generating the enactment effect, supporting and extending the theory of motor information reactivation.

## Data Availability

The raw data supporting the conclusions of this article will be made available by the authors on request.

## Abbreviations

The following abbreviations are used in this manuscript:

EEG	Electroencephalography
ERP	Event-related potential
FMRI	Functional magnetic resonance imaging
MEG	Magnetoencephalography
SPT	Subject-performed task
VT	Verbal task
Act-in	Activation-Integration

## Appendix A. Experimental Materials

The verb–noun phrases used in the experiment are presented below. The original stimuli were presented in Chinese; English translations are provided for reference.

**Chinese Phrase**	**English Translation**	**Degree of Enactability**
拧瓶盖	Twist a bottle cap	High
抛硬币	Toss a coin	High
下棋	Play chess	High
拍照	Take a photo	High
捡垃圾	Collect trash	High
翻书	Turn pages of a book	High
拿杯子	Hold a cup	High
划火柴	Strike a match	High
摇铃铛	Ring a bell	High
夹菜	Pick up food with chopsticks	High
弹钢琴	Play the piano	High
剪纸	Cut paper	High
浇花	Water flowers	High
扇风	Fan the air	High
打电话	Make a phone call	High
擦黑板	Wipe the blackboard	High
画圆	Draw a circle	High
喷香水	Spray perfume	High
喝水	Drink water	High
参军	Join the army	Low
登场	Make an entrance	Low
报恩	Repay a kindness	Low
乘飞机	Take a plane	Low
过关	Pass a level	Low
做生意	Run a business	Low
求学	Pursue academic studies	Low
拼音	Spell in pinyin	Low
让步	Concede ground	Low
按时	Meet the deadline	Low
押题	Predict exam questions	Low
分家	Split up a family	Low
发芽	Sprout a bud	Low
争气	Strive for success	Low
公布	Announce the result	Low
卡壳	Get stuck	Low
扛事	Shoulder responsibilities	Low
抽样	Conduct sampling	Low
投档	Submit application files	Low
